# Supplementary material for: Understanding water transport through graphene-based nanochannels via experimental control of slip length
Source: Nat Commun. 2022 Sep 28;13:5690. doi: 10.1038/s41467-022-33456-w (PMC9519754; doi:10.1038/s41467-022-33456-w)
Supplement: Supplementary file 2 — Supplementary Information [file 41467_2022_33456_MOESM2_ESM.pdf]

Supplementary Information

Understanding water transport through graphene-based nanochannels via experimental control of slip length

Wen et al

Supplementary Note 1: **Interlayer space of cation intercalated GOMs**

In this work, we prepared cation intercalated GOMs with two methods, membrane intercalation (X-M-GO) and solution intercalation (X-S-GO). The two preparation methods for the GO membrane led to a similar trend in the interlayer spacing when altering the intercalated cation (Supplementary Fig. 1). For both X-M-GO and X-S-GO, the interlayer space increases with the increased hydrated diameter of intercalated cations. In the case of X-M-GO, the interlayer space of K^+-M-GO (red column) is lower than pure GOM (short-dashed line), whereas Na^+-, Mg^{2+}- and Ca^{2+}-M-GO show larger interlayer space. This unique behaviour of K^+-M-GO is in close agreement with the previous research, attributing the reduced interlayer space to a squeezed hydration structure of intercalated cations[1]. However, the interlayer space of all X-S-GO showed reduced interlayer space compared to pure GO (blue columns), suggesting the structural distortion occurs to not only K^+ but also Na^+, Mg^{2+}, and Ca^{2+} cations[1]. This distortion could result from severely squeezed hydration structure during vacuum filtration and consequently the decreased interlayer space for X-S-GO.

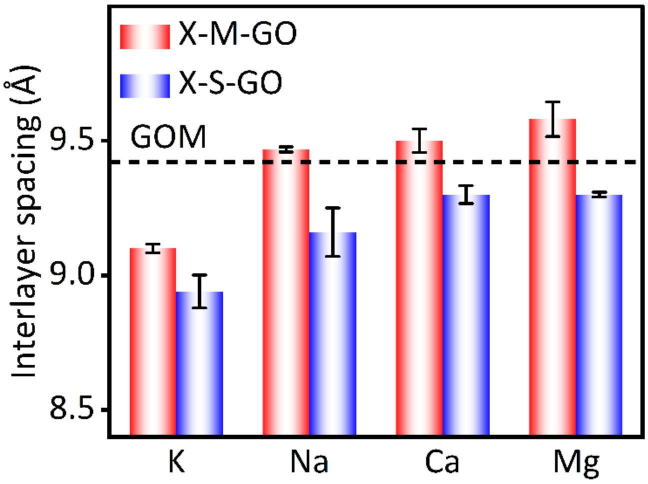

Supplementary Fig. 1: Interlayer space of cation intercalated GOMs in dry state. Here X stands for corresponding cation, and M and S indicate that the intercalation occurs with GOM and GO solution, respectively. The short-dashed line corresponds to the interlayer space of pure GOMs. Error bars are standard deviations of interlayer spaces from three membranes. Source data are provided as a Source Data file.

Supplementary Note 2: Water transport through nanochannels

We calculated the slip length (l_s) to explain the water transport mechanism through cation intercalated GOMs. The slip length is often used to characterize the friction interaction between water molecules and channel walls[2]. As shown in Supplementary Fig. 2a, when the water molecules transport through the channel, the channel wall interacts with the neighboured water molecules. The velocity of water molecules that interact with channel wall are impeded (white arrows in Supplementary Fig. 2a) while the water molecules in the middle of tunnel are unimpeded (black arrows in Supplementary Fig. 2a). Here, we plot a dashed straight line in Supplementary Fig. 2a on the left that corresponds to the plane of zero-velocity and dashed curve on the right indicating the plane of real-velocity. The tangential extension of the curve intercept with the straight line. This intercept is the extrapolated zero-velocity point. According to the physical definition, the slip length (l_s) is the distance from the channel wall to the extrapolated zero velocity point[3,4]. If the water molecules move frictionless through the channel (Supplementary Fig. 2b), the zero-velocity plane is parallel to the real velocity plane, resulting in infinitive slip length ($l_s = \infty$). Moreover, if the friction between water molecules and tunnel walls increased to maximum, the velocity of water molecules that are close to the tunnel walls approaches to zero (Supplementary Fig. 2c). The intercept point is located on the surface of the tunnel wall leading to a non-slip condition ($l_s = 0$).

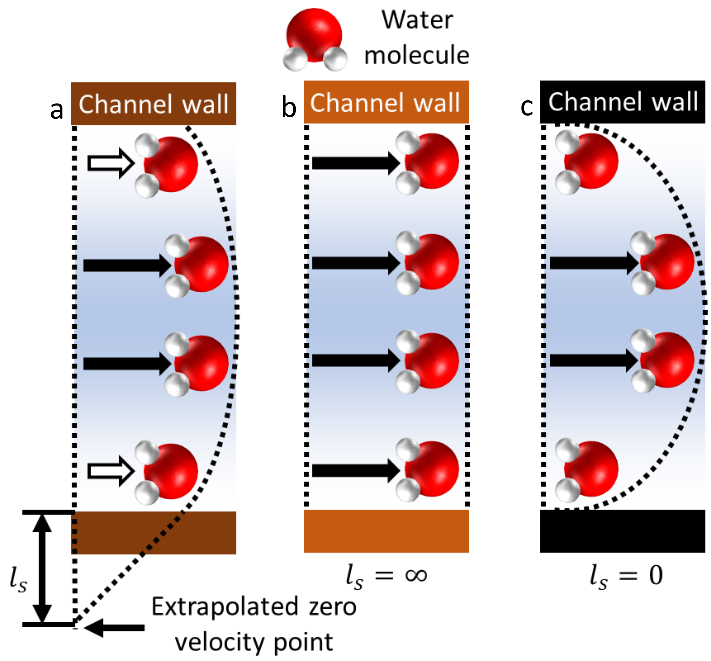

Supplementary Fig. 2: Schematic illustration of water transport through tunnels. a

Schematic of slip length (l_s). **b** Water transport frictionless through channel walls. The slip length is infinite ($l_s = \infty$). **c** Zero velocity of water molecules near to the channel walls showing non-slip condition ($l_s = 0$). Black and white arrows correspond to the unimpeded and impeded velocity of water transport, respectively.

According to Nair et al[5], the water transport through GO laminates fits the above illustration in Supplementary Fig. 2. Here we analysed the friction behaviour on water-GO flakes interface by calculating the slip length (l_s) of water transport through different cation intercalated GOMs.

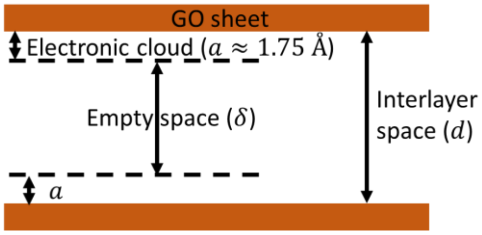

Supplementary Fig. 3: Schematic illustration of 'empty' space between GO sheets. The empty space equals to the interlayer space of GO minus two of the electronic cloud of graphene sheets.

We first calculate the 'empty' space (δ) for water transport (Supplementary Fig. 3),

$$\delta = d - 2a \tag{1}$$

where d is the interlayer space derived from XRD measurements of the respective GO membrane after water immersion and a is electronic cloud around graphene sheets (a≈1.75 Å)[5].

The total length (Supplementary Fig. 4) of water transport (l) is

$$l = \frac{hL}{d} \tag{2}$$

where h is the thickness of the membrane (h≈200 nm) and L is GO flake size (L≈480 nm)[5].

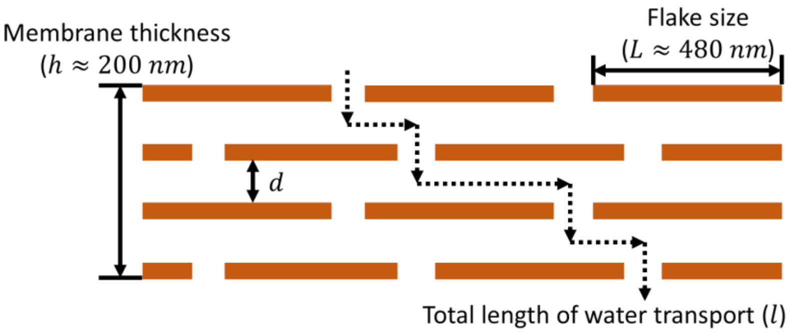

Supplementary Fig. 4: Schematic illustration of water transport pathway.

The theoretical water flux (Q) is

$$Q \approx \delta^3 \times \frac{1}{12\eta} \times \frac{1}{L} \times \frac{\Delta P}{l} \times \rho \tag{3}$$

where η is viscosity of bulk water (η=1 mPa·s), ρ is density of water (ρ=1 g/mL) and ΔP is driving pressure (ΔP=10^3 bar)[5].

Considering the experimental flux (J), the theoretical flux (Q) is enhanced by a factor (Θ) of

$$\Theta = \frac{J}{Q} \tag{4}$$

The slip length (l_s) is calculated from[5]

$$l_s \approx \frac{\Theta \times \delta}{8} \tag{5}$$

Supplementary Note 3: Carbon/cation ratio

The carbon-cation ratio was calculated as follows. According to our XPS results showing the C/O atomic ratio is 2.97 (Fig. 1g), we could estimate the number of moles of carbon in the 0.5 mg of GOM is

$$12\, mg/mmol \times n(C) + 16\, mg/mmol \times n(O) \approx 0.5\, mg \tag{6}$$

$$n(C)/n(O) = 2.97 \tag{7}$$

where n(C)≈28.7 µmol (here we made assumption that the contribution of H element to mass of GOM is negligible). Compared with the intercalated cations (0.2 µmol), the number of carbon atoms are ~144 times more than cations. This means that very limited number of cations existed in the nanochannels, indicating weak the steric hindrance of cations.

Supplementary Note 4: Steric hindrance

The steric hindrance of intercalated cations refers to the physical presence of the cations in between the free space of GO layers. It may slow down the water transport through the GO nanochannels. The steric hindrance caused by intercalated cations is related to the structure of non-hydrated cations, i.e., the ionic diameter. We plot the flux of X-S-GOMs with 0.2 μmol cation against the ionic diameter (D_I) as shown in Supplementary Fig. 5a. The largest K^+ (i.e., highest steric hindrance) show highest water flux. This is irrational that more hindrance to water transport results in higher water flux. Similar trend of slip length is shown in Supplementary Fig. 5b, where K^+ ions result in largest slip length (i.e., lowest water transport hinderance). Therefore, we exclude the effect of steric hindrance of cations to reduced water flux and slip length.

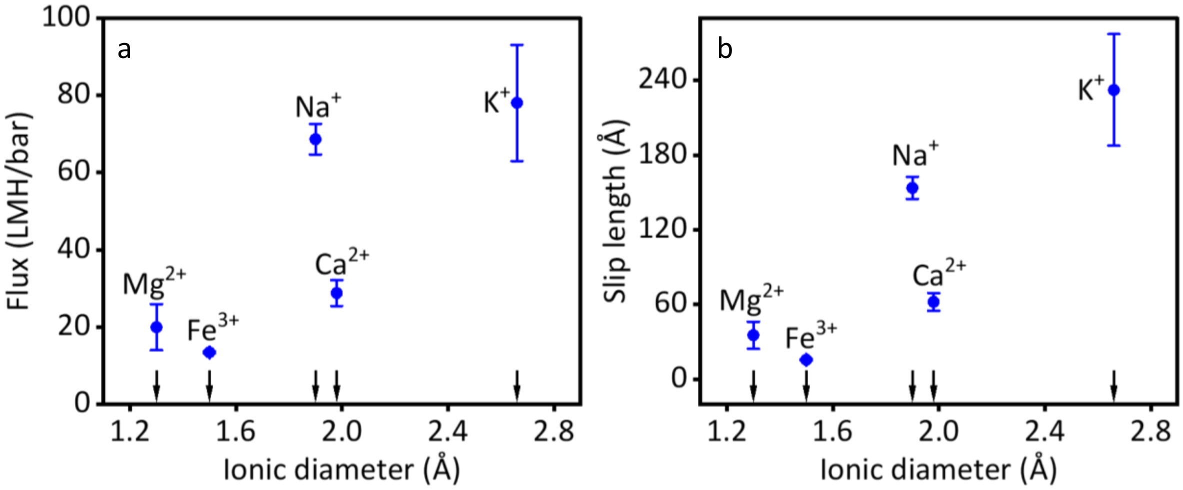

Supplementary Fig. 5: Steric hindrance affecting the water transport through 0.2 μmol of cation intercalated GOMs. a Variation of water flux of cation intercalated X-S-GO against ionic diameter of cations. **b** Variation of slip length with ionic diameter of cations. Error bars indicate the standard deviation from three tested membranes. Source data are provided as a Source Data file.

Supplementary Note 5: Cation intercalation stability test

To exclude differences in cation intercalation stability, we examined the integrity of GOMs and the stability of intercalated cations. The cation intercalated GOMs were cut into ~0.5 cm^2 triangle shape pieces and immersed into 5 mL of deionized water in small centrifuge tubes. The membranes were ultrasonicated for 0.5 h after immersion for 7 and 14 days. Photographs of GOMs pieces after ultrasonication were taken to examine whether they were peeled off from substrate or any cracks were created. As shown in Supplementary Fig. 6, these GOMs showed excellent attachment to PVDF substrate after soaking in water for 7 days and 14 days. No visible cracks were found on the membrane surface.

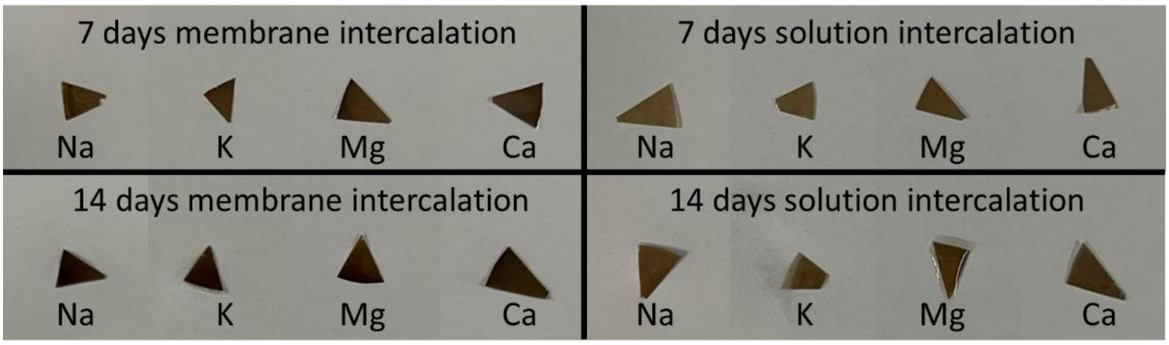

Supplementary Fig. 6: Visual presentation of cation intercalated GOM for integrity check after 7 days and 14 days. The membrane pieces were ~0.5 cm^2 in triangle shape. After 7 days and 14 days water soaking, the membranes were ultrasonicated for 0.5 h.

We also performed pressure driven filtration to study the intercalation stability. Deionized water was filtered through intercalated GOMs under ~0.9 bar of vacuum pressure for 5 h. Every 1 hour, we collected the water samples from permeate side. The volumes of these samples were measured for subsequent flux calculation. Concentrations of cations in the permeate solutions were measured by ICP-OES to calculate masses of eluted salt (NaCl, KCl, $MgCl_2$ and $CaCl_2$). As shown in Supplementary Fig. 7 for the volumes of permeate samples in each hour, pure GOMs had highest volume of permeation compared with intercalated GOMs in every single hour.

The ICP-OES results of each water samples were listed in Supplementary Table 1 for X-M-GO and Supplementary Table 2 for X-S-GO. The detection limit of ICP-OES instrument is 10 µg/L. We found the elution of cations were highest in the first hour of filtration and rapidly fell in the second hour. The cation concentrations were undetectable in the 5th hour for all samples.

Based on the above results, we could calculate the masses of eluted NaCl, KCl, $MgCl_2$ and $CaCl_2$. For example, the permeate volume of Na-GOM is 14.4 mL (from Supplementary Fig. 7a first hour) and the concentration of eluted Na^+ is 11.7 µg/L (from Supplementary Table 1 first hour). We could calculate the mass of eluted Na^+ is 11.7 µg/L*14.4 mL/1000=0.17 µg. Considering the mass ratio of Na^+ in NaCl is 23.0 g*mol^{-1}/58.4 g*mol^{-1}=0.39, the eluted mass of NaCl is 0.17 µg/0.39=0.4 µg. We calculated the masses of eluted NaCl, KCl, $MgCl_2$ and $CaCl_2$ and listed in Supplementary Table 3.

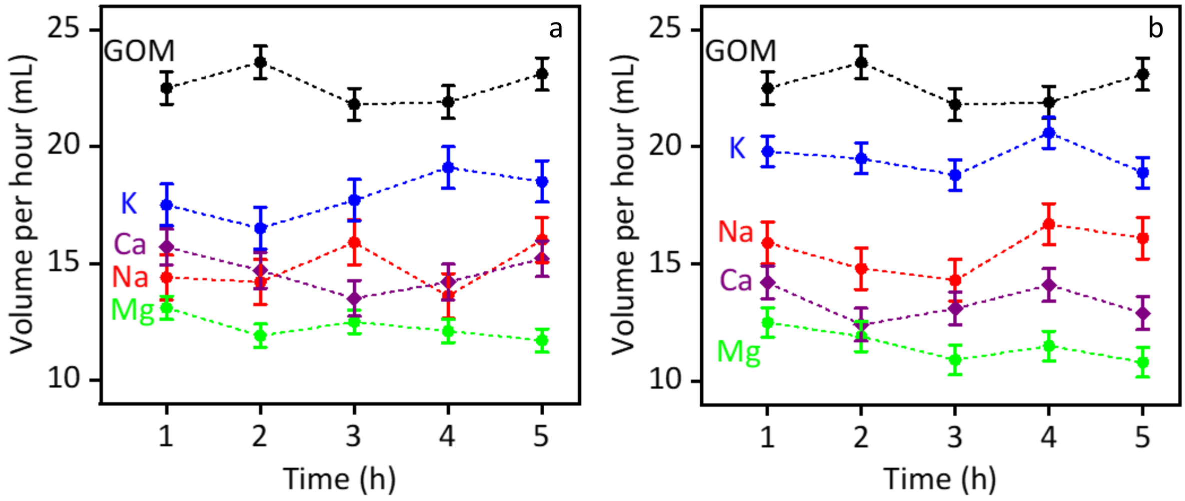

Supplementary Fig. 7: Volume of deionized water filtration through pure GOMs and cation intercalated GOMs in each hour. a X-M-GO and **b** X-S-GO. Error bars were the standard deviation from three independent samples. Source data are provided as a Source Data file.

Furthermore, we also tested the residue solution after solution intercalated GOMs preparation. We found the concentration of all residue solution were lower than the detection limit of ICP-OES, indicating adequate reaction of cations with GO suspension.

Supplementary Table 1: Concentration of eluted cations in each hour from X-M-GO

Time (h)	Na^+ Concentration (µg/L)	K^+ Concentration (µg/L)	Mg^{2+} Concentration (µg/L)	Ca^{2+} Concentration (µg/L)
1	11.7	73.4	29.9	41.5
2	10.0	12.0	21.5	19.1
3	N/A*	10.0	10.0	10.0
4	N/A	N/A	N/A	N/A
5	N/A	N/A	N/A	N/A

*N/A corresponds to the concentration is lower that detection limit of ICP-OES.

Supplementary Table 2: Concentration of eluted cations in each hour from X-S-GO

Time (h)	Na^+ Concentration (µg/L)	K^+ Concentration (µg/L)	Mg^{2+} Concentration (µg/L)	Ca^{2+} Concentration (µg/L)
1	56.1	94.4	30.5	44.4
2	10.0	29.8	21.9	20.3
3	N/A	10.9	10.0	10.0
4	N/A	10.0	N/A	N/A
5	N/A	N/A	N/A	N/A

Supplementary Table 3: Eluted salts mass from cation intercalated GOMs

Time (h)	Eluted NaCl (µg)		Eluted KCl (µg)		Eluted $MgCl_2$ (µg)		Eluted $CaCl_2$ (µg)	
	X-M-GO	X-S-GO	X-M-GO	X-S-GO	X-M-GO	X-S-GO	X-M-GO	X-S-GO
1	0.4	2.3	2.5	3.6	1.6	1.5	1.8	1.8
2	0.4	0.4	0.4	1.1	1.0	1.0	0.8	0.7
3	N/A	N/A	0.3	0.4	0.5	0.4	0.4	0.4
4	N/A	N/A	N/A	0.4	N/A	N/A	N/A	N/A
5	N/A	N/A	N/A	N/A	N/A	N/A	N/A	N/A
Total	~0.8	~2.6	~3.2	~5.5	~3.1	~3.0	~3.0	~2.8

Supplementary Note 6: Simulation study on water affinity towards cation intercalation at functionalized graphene

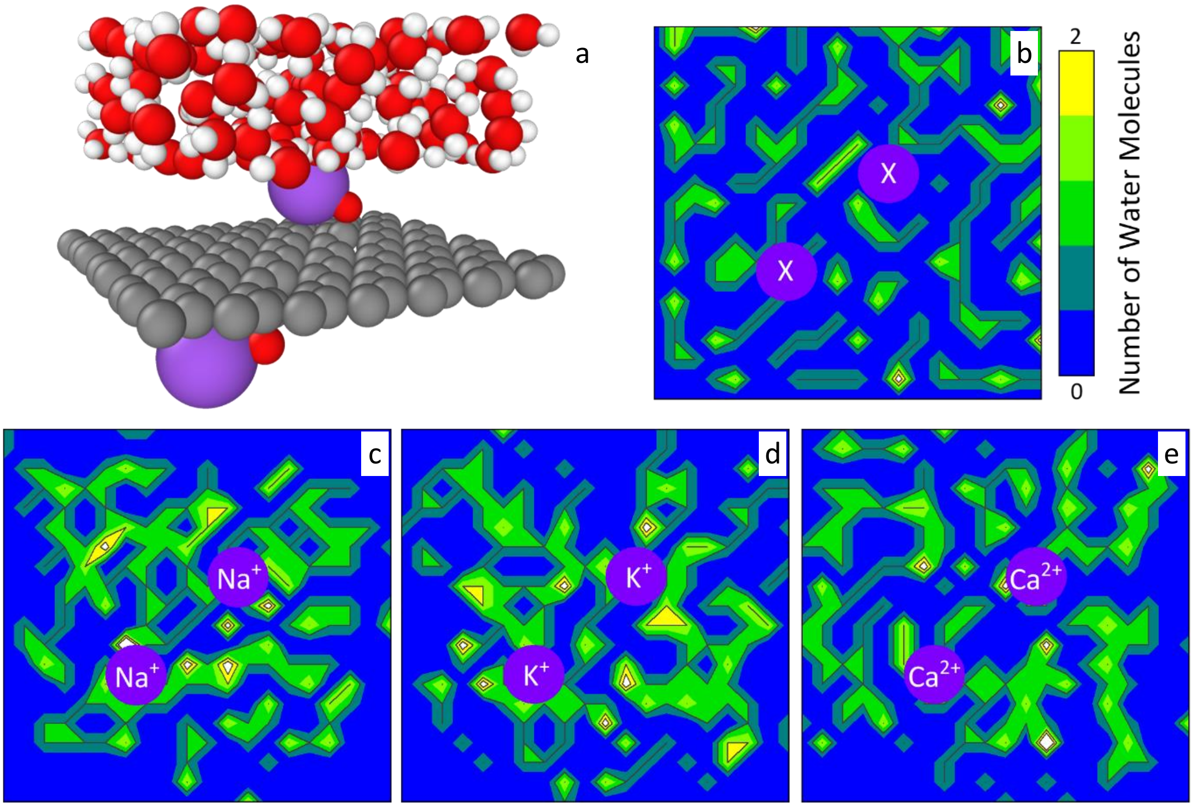

Supplementary Fig. 8: Simulation study on the water affinity towards cation intercalation at graphene functionalised with hydroxy group. a Schematic diagram of the simulation cell at initial state (0 ps). The supercell consists of a graphene sheet (grey spheres) functionalised with hydroxy groups intercalated with a cation (violet and red spheres correspond to cations and oxygen atoms from hydroxy groups). The supercell is filled with water molecules (red and white spheres are oxygen and hydrogen atoms, respectively). The periodic boundary conditions ensure the nanoconfinement. **b** Distribution of water molecules at the initial state. Violet circles are the position of cations. X is a placeholder for different types of cations. The Blue area represents a water-free area, while from cyan to red corresponded to an increased number of water molecules accumulated. **c-e** Distribution of water molecules after 4 ps for functionalized graphitic sheets intercalated with K^+, Na^+ and Ca^{2+}, respectively. Source data are provided as a Source Data file.

Previous studies suggest that cations intercalate GO mainly via cation-π interaction[6,7]. It may also occur at *sp*^{3} carbon areas, where the epoxy, hydroxy and carboxy groups exist. Such intercalation may affect the slip length when water molecules transport through GO membranes. The carboxy groups, located at the edges of GO sheets[8], have limited contribution to the morphology of the GO nanochannels. Therefore, the cation intercalation with carboxy groups may not affect the slip length. On the other hand, hydroxy and epoxy groups are located at basal plane of GO sheets which may affect the slip length. To investigate

this, we performed further simulation study using cation-hydroxy intercalation. As shown in Supplementary Fig. 8a, the simulation cell was chosen to emulate the cation-hydroxy interaction. Two cations were attached to the hydroxy groups on the graphene basal plane. Periodic boundary conditions were applied for bilayer configuration. The simulation cell was filled with water molecules. The projection of water molecules on the plane was examined at initial state (Supplementary Fig. 8b) and final state (Supplementary Fig. 8c-e) after relaxing the cell for 4 ps. Mg atom failed to bind with hydroxy groups similar as the case for graphitic areas.

We observed that the water molecules are rearranged and become denser due to the attraction of positively charged cations. However, unlike the case of cation-π interaction (Fig. 4c-e), the density of water molecules around the Na^+, K^+ and Ca^{2+} ions show no obvious difference in water affinities (Supplementary Fig. 8c-e). Based on that we believe that the cation intercalation with *sp*^{3} carbon area cannot explain the trend of the slip length observed in Figure 2D. As the water affinity shows a matching trend if the cations interact with the *sp*^{2} carbon (Fig. 4c-e), it suggests that the cation-pi interaction is the more dominant case for governing the slip length.

Supplementary References

1. Chen, L. et al. Ion sieving in graphene oxide membranes via cationic control of interlayer spacing. Nature **550**, 380–383 (2017).
2. Qin, X., Yuan, Q., Zhao, Y., Xie, S. & Liu, Z. Measurement of the rate of water translocation through carbon nanotubes. Nano Lett **11**, 2173–2177 (2011).
3. Myers, T. G. Why are slip lengths so large in carbon nanotubes? Microfluidics and Nanofluidics 2010 10:5 **10**, 1141–1145 (2010).
4. Radha, B. et al. Molecular transport through capillaries made with atomic-scale precision. Nature **538**, 222–225 (2016).
5. Nair, R. R., Wu, H. A., Jayaram, P. N., Grigorieva, I. v & Geim, A. K. Unimpeded permeation of water through helium-leak–tight graphene-based membranes. Science (1979) **335**, 442–444 (2012).
6. Sun, P. et al. Selective ion penetration of graphene oxide membranes. ACS Nano **7**, 428–437 (2013).
7. Sun, P. et al. Selective trans-membrane transport of alkali and alkaline earth cations through graphene oxide membranes based on cation− π interactions. ACS Nano **8**, 850–859 (2014).
8. Lerf, A., He, H., Forster, M. & Klinowski, J. Structure of graphite oxide revisited. J Phys Chem B **102**, 4477–4482 (1998).
